# Descending necrotizing mediastinitis caused by *Streptococcus constellatus*: A case report and review of the literature

**DOI:** 10.1097/MD.0000000000033458

**Published:** 2022-04-07

**Authors:** Jian Guo, Liheng Lin, Haoge Zhou, Wenfan Yang, Sen Shi

**Affiliations:** a Department of Vascular Surgery, The Affiliated Hospital of Southwest Medical University, Luzhou, China; b Department of Anesthesiology, The Affiliated Hospital of Southwest Medical University, Luzhou, China.

**Keywords:** descending necrotizing mediastinitis, sepsis, *Streptococcus constellatus*

## Abstract

Descending necrotizing mediastinitis (DNM) is a rare but severe mediastinal infection. If not diagnosed and treated promptly, the consequences can be very serious. Here, we shared a successful diagnosis and treatment case of DNM that originates from oral to neck and mediastinum caused by *Streptococcus constellatus (S constellatus*). *S constellatus* is a clinically uncommon gram-positive coccus and is known for its ability to form abscesses. Timely surgical drainage and the correct use of antibiotics are key to successful treatment.

**Patient concerns:**

A 53-year-old male admitted to hospital with painful swelling of the right cheek, persistent oral pus and moderate fever lasting 1 week, followed by rapid development of a mediastinal abscess.

**Diagnoses:**

He was diagnosed with DNM caused by *S constellatus*.

**Interventions:**

On the evening of admission, an emergency tracheotomy and thoracoscopic exploration and drainage of the right mediastinum, floor of the mouth, parapharynx and neck abscess were performed. Antibiotics were administered immediately.

**Outcomes:**

At 28 days post-operatively, the abscess was absorbed, bilateral lung exudate decreased and the patient temperature, aspartate transaminase, alanine transaminase, bilirubin and platelets returned to normal. The patient was discharged after completing 4 weeks of antibiotic therapy. Follow-up at 3 months after discharge revealed no recurrence of the abscess.

**Lessons:**

Early surgical drainage and antibiotics treatment are important in mediastinal abscesses and infectious shock due to Streptococcus asteroids.

## 1. Introduction

Descending necrotizing mediastinitis (DNM) is a serious mediastinal infection that originates from oral or cervical infection. Those occurring in the upper mediastinal space above the bulge are called Localized DNM-type I. If the infection extends into the mediastinum, it is called Diffuse DNM-type II.^[1]^ Bacteria that frequently cause DNM include anaerobic organisms and gram-negative pathogens. However, when the clinical condition of a patient diagnosed with DNM deteriorates rapidly, infection with unconventional microorganisms such as *Streptococcus constellatus (S constellatus*) must be considered.

*S constellatus* is one of the *Streptococcus anginosus* group (SAG, formerly Streptococcus milleri), usually present in the normal flora of the human oral cavity, urogenital region, and intestinal tract.^[2]^ It can cause rapidly progressing and life-threatening descending infections that can create extensive local abscesses, and cause pneumonia and septic shock. Here we reported a case of diffuse descending infection in the maxillofacial region, neck, and mediastinum caused by *S constellatus* that progressed aggressively and quickly. This report will improve our understanding of *S constellatus*-associated infections and provide new perceptions into the management and treatment of severe life-threatening infections.

## 2. Case presentation

**Figure 1.**
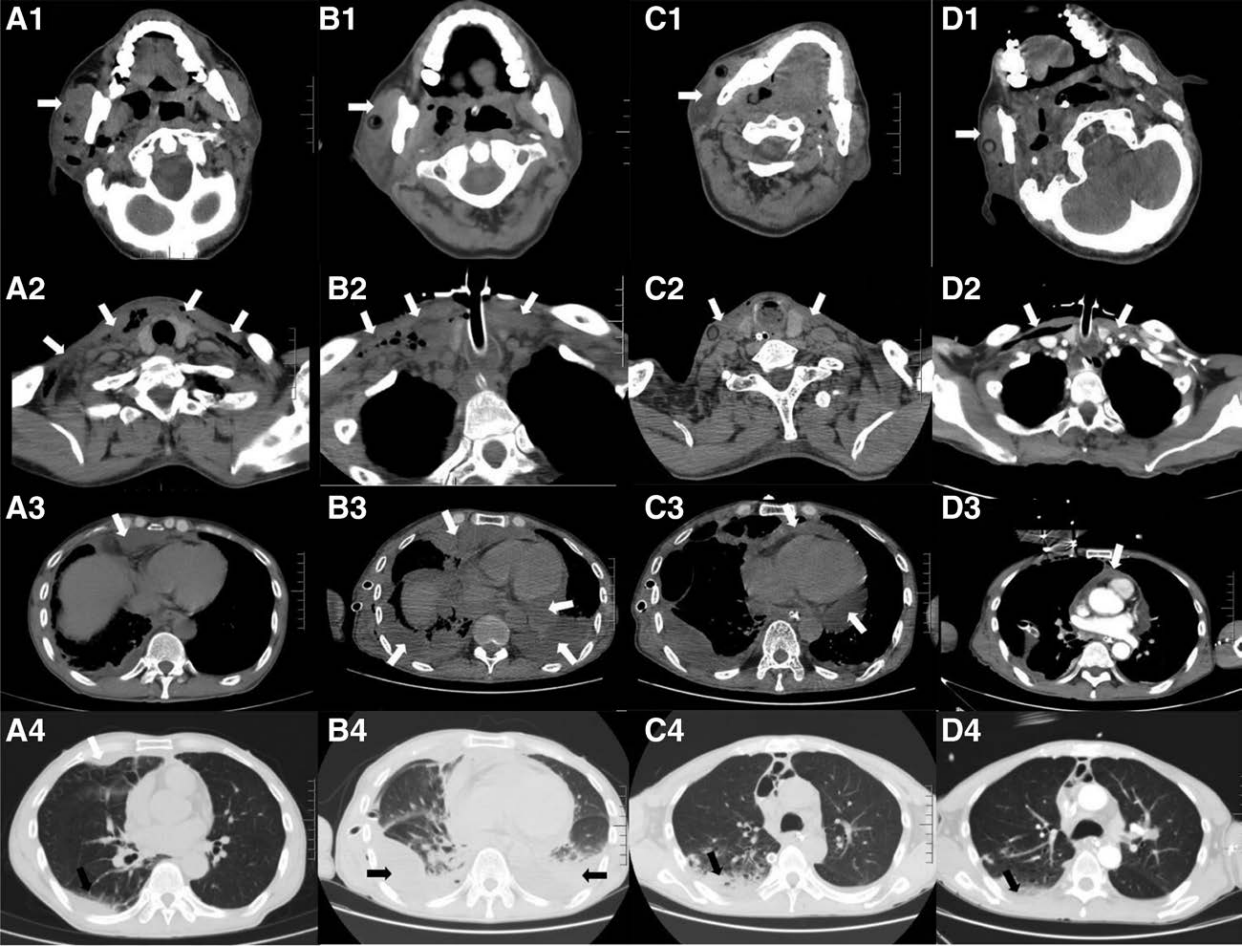
Head and chest CT images at different time-points. (A) CT images at admission; (B) CT images after surgery; (C) CT images after surgical and antibiotic treatment; (D) CT images at discharge. White arrow indicates the abscess, effusion, and pneumatization. Black arrow indicates the lung exudation. CT = computed tomography.

A 53-year-old male was admitted to the hospital because of right cheek swelling with pain, persistent pus from his oral cavity along with moderate fever that developed 1 week. Prior to admission to our hospital, he had taken oral metronidazole for 3 days, but then his condition worsened with dyspnea and chest pain. On admission, the patient was apathetic and somnolent. His temperature was 38.8°C, pulse rate 129 beats/min, respiratory rate 23 breaths/min, blood pressure 87/46 mm Hg, and oxygen saturation 95% in ambient air. Physical examination revealed severe right facial edema (measured about 8 cm × 10 cm), restricted mouth open ability (1 transverse finger), and bilateral neck swelling, with elevated skin temperature, increased tension, tenderness, and redness. His bilateral temporomandibular joint function was limited as well. Oral examination showed several dental caries, and the rest of the oral cavity could not be observed due to the limited mouth open ability. A lung examination revealed reduced breath sounds in both lungs, but no obvious moist rales were noted. Computed tomography (CT) revealed extensive swelling and pneumatization in the areas including the right maxillofacial, oropharyngeal, laryngopharyngeal, submandibular, bilateral cervical, and mediastinum (Fig. 1A1–4). Infection in the right lung and double pulmonary emphysema signs can be seen. Laboratory results showed a normal leucocyte count [7.02 × 10^9^/L; normal range, (3.5–9.5) × 10^9^/L], a high neutrophil percentage (90.30%; normal range, 40%–70%), a significantly high procalcitonin level (6.530 ng/mL; normal range, <0.5 ng/mL), an elevated lactic acid level (6.34 mmol/L; normal range, 0.5–1.7 mmol/L), a high level of C-reactive protein (194.5 mg/L; normal range, 1–8 mg/L). His wife reported that he did not have history of infectious diseases, respiratory diseases, diabetes mellitus, or hypertension, and denied recent travel, surgery, or trauma. The initial diagnoses were multi-space infection of the floor of the mouth, mediastinal infection, and septic shock.

Based on the patient critical condition, he was empirically treated with meropenem and underwent an urgent tracheotomy and thoracoscopic exploration and drainage of abscesses in the right mediastinum, floor of the mouth, parapharynx, and neck on the night of admission. Gram-positive cocci were simultaneously detected from the patient blood and then identified as *S constellatus* on day 2. No pathogen was detected in the culture of mediastinal abscess fluid and sputum samples. Drug sensitivity tests showed that *S constellatus* was sensitive to meropenem, penicillin G, ampicillin, ceftriaxone, cefuroxime, cefepime, cefotaxime, linezolid, daptomycin, levofloxacin, erythromycin, clindamycin, vancomycin, and tetracycline. Therefore, vancomycin was added to meropenem.

**Figure 2.**
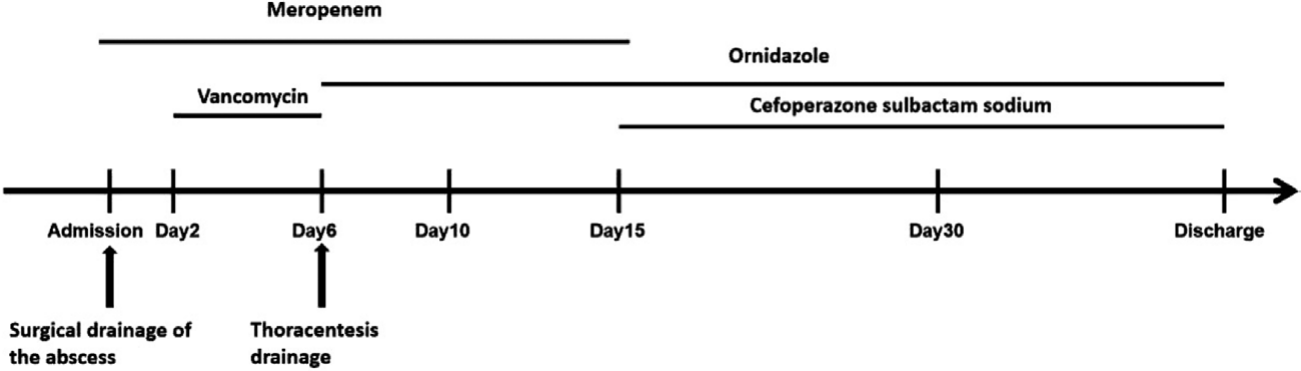
The antibiotic treatment timeline.

Five days later, chest CT suggested additional medium amount of pericardial effusion, aggravated lung exudation, enlarged scope of bilateral pleural effusion, and increased gas accumulation in the neck and mediastinum (Fig. 1B1–4). Besides, the patient aspartate transaminase, alanine transaminase, and bilirubin were increased and the leucocyte count progressively elevated to 20.22 × 10^9^/L. Considering the specificity of the infection in the floor of the mouth and the possibility of gas production due to anaerobic bacterial infection, the antimicrobial regimen was changed to ornidazole and meropenem. Thoracentesis drainage was performed subsequently. During this period, the patient showed a progressive increase in platelets up to 800 × 10^9/L. We considered this to be infection-related and added antiplatelet therapy with aspirin beside the antibiotics. On day 4 after the change of antibiotics, the inflammatory indicators decreased and the temperature started to fall. Five days later, meropenem treatment had been administered for 2 weeks, and chest CT showed a decrease in the accumulation of fluid in the neck, mediastinum, and pleural cavity (Fig. 1C1–4). The infection was improved, so the antibiotics were adjusted to cefoperazone sulbactam sodium and ornidazole. The antibiotic treatment timeline was showed in Figure 2.

Twenty eight days after surgery, chest CT showed that, compared to the previous one, the abscess was absorbed, bilateral lung exudation was reduced and there was a little air accumulation in the maxillofacial and cervical areas and a small amount of pericardial effusion (Fig. 1D1–4). The patient body temperature, aspartate transaminase, alanine transaminase, bilirubin, and platelets returned to normal as well. After completing a 4-week course of antibiotic therapy, the patient was discharged to a rural general hospital in a stable condition. He was discharged after another 2 weeks of antibiotic treatment successfully. Follow-up at 3 months after discharge showed no recurrence of the abscess.

## 3. Discussion

*S constellatus* belongs to the SAG, which was also known as the Streptococcus Milleri Group. SAG consists of 3 Gram-positive commensal species: *S anginosis, S constellatus*, and *S intermedius*. SAG is a commensal organism of the human body, widely distributed in the oral cavity, nasal cavity, throat, gastrointestinal tract, and urogenital tract. *S anginosis* was the most common species in gastrointestinal and genitourinary tracts, while *S constellatus* was the most common species in respiratory specimens.^[3]^ This group of bacteria does not grow well in aerobic environments and needs to be placed in 5% CO_2_ or anaerobic environments to grow.^[4]^ This leads to them being easily missed in the microbial culture tests performed in aerobic environments. Infections caused by this pathogen may be overlooked by clinicians as well. Some studies showed that metagenomic next-generation sequencing could be used to detect pathogens in a target-independent manner.^[5]^ Next-generation sequencing (NGS) is the process of randomly amplifying, fragmenting, and purifying deoxyribonucleic acid (DNA) to construct DNA libraries, and by performing extension reactions on tens of thousands of clones in the library, high-quality sequencing data is finally generated. Then, the sequencing data are aligned to the bacterial, virus, and fungal databases to determine whether certain pathogens have been infected. Although NGS can sequence thousands to billions of DNA fragments simultaneously and rapidly identify pathogens, it has its own drawbacks. First, NGS is not absolute in detecting pathogenic bacteria and there is also a possibility of missing detection.^[6]^ Second, if a positive result is found, a drug sensitivity test cannot be performed to clarify the drug susceptibility of the organism compared to traditional pathogenic tests such as blood and sputum cultures. Third, the NGS is relatively expensive. This patient blood culture on the first day has already detected *S constellatus*, helping us make treatment more precise and timely and reduce antibiotic abuse.

*S constellatus* used to be considered an opportunistic pathogen, causing infections in patients with chronic obstructive pulmonary disease, diabetes mellitus, cardiovascular diseases, alcoholism, and immunosuppression. Now, more and more diseases have been identified as potential predisposing factors, including chest trauma, chest surgery, uremia, collagen vascular disease, preexisting pericardial disease, and malignancy. Recent studies have found that *S constellatus* usually exacerbates inflammation in association with other anerobic organisms and gram-negative pathogens, especially in healthy individuals^.[7,8]^ Treatment with broad-spectrum antibiotics and anti-anaerobic antibiotics is essential. Our patient had neither predisposing factors nor an underlying disease as described above and his blood culture, sputum culture, and pus culture showed no anaerobic bacteria. However, his condition improved significantly after treatment with additional anti-anaerobic drugs. The possibility of anaerobic bacterial infection cannot be excluded even in the absence of a positive culture, due to the difficulty of culturing anaerobic bacteria. This treatment offered fresh insights on *S constellatus*-associated clinical treatment management.

The distinctive feature of *S constellatus* is its abscess-forming capability. These abscesses can spread hematogenously, leading to metastatic abscesses in various organs and bacteremia, or can be caused by direct spread through adjacent infected lesions. In this case, the infection in the oropharyngeal region descended into the mediastinum through the deep cervical and superficial fascial surfaces. We searched PubMed database for cases of *S constellatus* over the last decade and found that almost cases reported abscess formation ranging from the brain, orbit, lung, and mediastinum to the heart, ovaries, kidney, liver, spine, and thighs.^[6–11]^ We complied 6 cases of DNM caused by *S constellatus*, and this was the second case of such severe, rapidly progressing septic shock (Table 1).^[11–16]^ In another case, which was also very aggressive, a patient developed necrotizing fasciitis in the maxillofacial region, neck, and upper mediastinum after experiencing cold and fatigue, followed by rapid decompensation with resulting septic shock caused by *S constellatus* and Klebsiella oxytoca. As we can see, the patients’ age ranged from 6 to 77 years old and the length of stay varied from 14 to 42 days. It appears that poor oral hygiene is probably the most likely cause of *S constellatus* infection. For most patients, the infection gradually spread from the oral cavity to the floor of the mouth, submandibular space, neck, chest, and mediastinum within a week. Fortunately, most of them were stable and all of them were eventually cured and discharged from the hospital. *S constellatus* is sensitive to a range of antibiotics and the recommended general duration of antimicrobial treatment is 4 to 6 weeks, particularly when there is abscess formation.^[16]^ Timely administration of sensitive antibiotics together with adequate drainage of the abscess is strongly recommended, which may prevent further deterioration and recurrence. Even in patients in poor general condition due to severe infection with DNM, peritonitis, or septic shock, early surgical drainage of the abscess with aggressive antibiotic treatment can completely control the patient condition with a relatively good prognosis.

**Table 1:** A brief on DNM caused by *S constellatus*.

Author/time	Age/se.	Underlying disease	Initial infection cites	Secondary infection sites	Clinical symptoms	Diagnostic basis	Pathogens	Drug treatment (time)	Surgery (time)	Outcome	Length of stay in hospital
Taisuke Kaiho, 2016	50/Female	Dental caries	Oral cavity	Neck, Mediastinum	Fever, Neck pain, swelling and stiffness, Dyspnea	Blood cultures	*S constellatus*	Meropenem (Not mention)	Emergency drainage of neck and mediastinum (Not mention)	Cure	28 d
Rui-hai Ye, 2020	49/Female	Dental caries Tuberculosis (cured)	Oral cavity	Mediastinum	Fever, Chest pain, Cough	Pus cultures	*S constellatus*	Mezlocillin/Sulbactam (2 wk)	EBUS-TBNA (Not mention)	Cure	14 d
Ling Jin, 2020	48/Male	Diabetes	Throat Tonsil	Neck, Mediastinum	Fever, Dysphagia, Throat pain, Neck pain and swelling	Pus cultures	Klebsiella oxytoca, *S constellatus*, Candida albicans, Candida guilliermondii	Meropenem Tigecycline Fluconazole (Not mention)	Tracheotomy, Neck incision and drainage (On the second d of admission)	Cure	37 d
Y M Bhatt, 2011	77/Male	None	Throat	Neck, Mediastinum	Throat pain, Dysphagia	Tissue cultures	*S constellatus*, Gram-negative anaerobic bacilli	Tazocin Clindamycin (3 wk)	Drainage of neck and mediastinum (Not mention)	Cure	42 d
Yongfei Zhang, 2022	6/Female	None	Oral cavity Throat	Mediastinum, Thoracic cavity	Fever, Odynophagia, Dental pain, Tachypnea, Chest pain	Pus cultures	*S constellatus*	Meropenem Linezolid (Not mention)	Ultrasound-guided thoracentesis (on the second d of admission) VATS for thoracic and mediastinal drainage (on the 4th day of admission)	Cure	18 d
Masahiro Oshima, 2011	61/Male	Dental caries and extracting a tooth	Neck	Mediastinum, Thoracic cavity	Neck swelling	Pleural effusion cultures	*S constellatus*	Not mention	Drainage of cervical abscess Thoracic drainage Thoracotomy (Not mention)	Cure	30 d

DNM = descending necrotizing mediastinitis, VATS = video-assisted thoracic surgery.

In this case, the patient had a very severe infection on admission, but the leucocyte count was not high. Normally, a portion of the leukocytes flow in the blood called the circulating pool, and another portion clings to the capillary walls, called the marginal pool. Leukocytes in the marginal pool cannot be detected by a leucocyte count. These 2 components, circulating pool and marginal pool, are in a dynamic balance. When the infection is severe, some leukocyte adhesion molecules and vascular endothelial cell adhesion molecules are activated by inflammatory mediators, making it easy for large numbers of leukocytes to adhere to the vessel wall and migrate across the endothelium to enter the tissue, ultimately leading to a decrease in leukocytes in the circulating pool.^[17]^ After anti-infective treatment, leukocytes can move rapidly from the marginal pool into the circulating pool, causing a significant increase in leukocyte count. In this case, after surgery and antibiotic treatment, the patient leukocytes were elevated and then gradually returned to normal levels after the infection was controlled. This patient also presented with abnormally elevated platelets. We considered this to be reactive thrombocytosis, seen in iron deficiency anemia, infections, and hyposplenism.^[18]^ Platelets are reduced to normal levels after anti-infective treatment. Previous studies have shown that *S constellatus* infections are often followed by abnormal liver function, which is consistent with the presentation of our patient.^[19]^

## 4. Conclusion

In general, *S constellatus* is a rare pathogen and can uncommonly cause mediastinal abscesses. Here, we presented a case of DNM caused by *S constellatus* infection with a very aggressive condition. DNM can become aggressive and even fatal if the infection and inflammation spread widely within the mediastinum due to the lack of timely surgical drainage. This case stressed the importance of early surgical drainage and the use of antibiotics in the treatment of mediastinal abscesses and septic shock caused by *S constellatus*, which can significantly reduce the complications, length of stay, and costs. Although some pathogens are uncommon in clinical practice, we still need to pay attention to such infections.

## Author contributions

**Data curation:** Jian Guo, Liheng Lin, Haoge Zhou.

**Writing – original draft:** Jian Guo, Liheng Lin, Sen Shi.

**Writing – review & editing:** Jian Guo, Wenfan Yang, Sen Shi.
